# Oculogyric crisis mimicked epilepsy in a Chinese aromatic L-amino acid decarboxylase-deficiency patient: A case report

**DOI:** 10.3389/fneur.2022.919583

**Published:** 2022-09-01

**Authors:** Hongmei Wang, Jiahong Li, Ji Zhou, Lifang Dai, Changhong Ding, Mo Li, Weixing Feng, Fang Fang, Xiaotun Ren, Xiaohui Wang

**Affiliations:** ^1^Department of Neurology, National Center for Children's Health, Beijing Children's Hospital, Capital Medical University, Beijing, China; ^2^Department of Gastroenterology, National Center for Children's Health, Beijing Children's Hospital, Capital Medical University, Beijing, China

**Keywords:** aromatic L-amino acid decarboxylase deficiency (AADCD), epilepsy, oculogyric crisis, DDC gene, developmental delay

## Abstract

**Background:**

Aromatic amino acid decarboxylase (AADC) deficiency is a rare, autosomal recessive neurometabolic disorder with heterogeneous phenotype, including hypotonia, movement disorders, autonomic dysfunction, and developmental delay. Here, we reported a Chinese patient with AADCD who was initially misdiagnosed with epilepsy.

**Case presentation:**

The proband was a 4-month-old Chinese girl, representing hypotonia, episodes of oculogyric crises with dystonia, and delayed developmental milestones. The patient was first misdiagnosed with epilepsy because of the similarity between episodes of oculogyric crisis and epileptic seizure. The accurate diagnosis of AADCD was established through analysis of neurotransmitters in cerebrospinal fluid (CSF). The genetic test confirmed the patient carried novel compound heterozygous mutations in the *DDC* gene:c.419G>A and c.1375C>T.

**Conclusion:**

This study reported a patient with AADCD who was initially misdiagnosed as epilepsy. Two novel missense mutations in the *DDC* gene were identified from the patient and her family. Little infants with epileptic-like attacks should consider AADCD. An accurate diagnosis of AADCD is essential for drug choice and patient management.

## Introduction

Aromatic L-amino acid decarboxylase deficiency (AADCD) (OMIM: 608643) is an autosomal recessive, neurometabolic disorder caused by disease-causing variants in the dihydroxyphenylalanine decarboxylase (*DDC*) gene. The *DDC* gene is located at chromosome 7p12.2-p12.3 and has 15 exons that encode aromatic L-amino acid decarboxylase (AADC), consisting of 480 amino acids (1, 2). The conformation of AADC is a homodimeric pyridoxal 5′-phosphate (PLP)-dependentalpha-decarboxylase, and each monomer is comprised of a large domain containing the PLP-binding site, a C-terminal small domain, an N-terminal domain located on the top of the large domain and a mobile loop important for catalytic activity (3). The AADC works as the final enzyme in the biosynthesis of serotonin and dopamine, which transforms L-dopa and 5-hydroxytryptophan (5-HTP) into dopamine and serotonin, respectively. Some residues were reported important for enzyme catalysis, including residues that participate in PLP binding, and the localization of substrate binding and cofactor stabilization (4). The deficiency of AADC results in a combined defect of serotonin, dopamine, norepinephrine, and epinephrine (5).

The phenotype of AADCD is highly heterogeneous, which can be explained by the deficiencies of different neurotransmitters. Dopamine deficiency compromises cognition, emotion, and voluntary movements. Norepinephrine and epinephrine deficiency affects attention, emotion, sleeping, and stress hormone levels. The reduction of serotonin influences memory, learning ability, emotion, cardiovascular function, and endocrine function (5). Overall, the key symptoms are clinically characterized as early-onset hypotonia, movement disorders (mainly oculogyric crises, dystonia, and hypokinesia), psychomotor developmental delay, and autonomic dysfunction (mainly ptosis and excessive sweating). These symptoms were reported in over 65% of patients (1, 6). Less-common neurologic symptoms include epileptic seizures, sleeping disorders, and some behavior problems, such as irritability, dysphoria, and excessive crying). Other non-neurologic symptoms are gastrointestinal problems and intermittent hypoglycemia, which are not AADCD specific (7). According to the consensus guidelines, there are three key diagnostic tests for AADCD: (1) analysis of neurotransmitter metabolite levels in cerebrospinal fluid (CSF); (2) *DDC* gene pathogenic variants, and (3) analysis of AADC enzyme activity in plasma (1). Worth mentioning is the neurological symptoms in AADCD that can be commonly seen in epilepsy or cerebral palsy, which may lead to delayed diagnosis or even misdiagnosis (8). Due to the rarity of AADCD, the experience of diagnosis and treatment is limited, and many patients' treatment responses are difficult to be predicted.

Here, we report a Chinese Mainland patient with AADCD caused by two novel compound heterozygous mutations in the *DDC* gene, and initially misdiagnosed as status epilepticus.

## Materials and methods

### Medical exome sequencing

Genomic DNA was extracted from peripheral blood using the Solpure Blood DNA Kit (Magen). Custom-designed NimbleGenSeqCap probes (Roche NimbleGen, Madison, WI, USA) were used for in-solution hybridization to enrich target sequences, which included coding exonic regions of approximately 5,177 OMIM-recorded genes and known pathogenic variants from deep intronic or other non-coding regions. DNA samples were indexed and sequenced on the AmCareSeq2000 (Amcare, Guangzhou, China). The average coverage depth was about 200 × with over 98% of the target regions covered by at least 20 reads. Sequenced reads were compared with the reference human genome version (GRCh37/hg19). Nucleotide changes found in aligned reads were pulled and analyzed through the NextGENe software (Version 2.4.2) (SofGenetics, State College, PA, USA). Sequence variants were annotated and included population (gnomAD, 1000 Genomes, dbSNP), and variant databases (Clinvar, HGMD). Online software Polyphen-2 and SIFT were used for *in silico* analysis of missense variants. Variants were classified into “Pathogenic”, “Likely Pathogenic”, “Uncertain Significance”, “Likely Benign”, or “Benign” according to the American College of Medical Genetics (ACMG) guidelines (9).

### Sanger sequencing

Sanger sequencing was performed to confirm the variants identified by MES. The primers were as follows:

chr7:50605574: F1 5′-ATTTTCCCAAGAGCTCGGTTTTG-3′ R1 5′-TCAGGCCTTTGAATCACATCTGA-3′ chr7:50530997: F2 5′-TTTTAAGGTGAGGGAAATGCACT-3′ R1 5′-CTTCTTCCTGATGTACGGTAGGG-3′

The reference sequence of *DDC* is NM_000790.4.

### Case presentation

The proband's data were acquired from The FUTang Updating medical REcords (FUTURE) Database (10). The proband was a female, the second child of a non-consanguineous couple. Both of the parents are healthy. Her mother was conceived through *in vitro* fertilization (IVF), and both pregnancy and delivery were uneventful. Her birth weight was 2.35 kg, and the Apgar score was 10 at 1 and 5 min.

After birth, she displayed weak sucking power and feeding difficulties. At 2.5-month of age, she first started to show eye deviation characterized by eyes turning up or upper right gazing, which is accompanied by stiff extension or flexion of the limbs. No cyanosis, incontinence, or salivation was observed during the breakout. At first, the episodes occurred 2–3 times per month, and the frequency eventually increased to 7–8 times per month. The duration of episodes usually lasted 5–6 min but, sometimes, can last up to 30 min. The episodes could relieve spontaneously and are usually provoked by crying, fatigue, or infection, which can be alleviated by relaxation or sleep. At 4 months of age, the episodes of eye deviation aggravated, 2–3 times per day and lasted for 0.5–2 h. She was then admitted to our hospital with epileptic status, and treated with anti-epileptic therapy in the emergency room. She was given antiepileptic drugs, including intravenous midazolam, diazepam, and vitamin B6 with limited efficacy. Physical examination showed a weight of 7.5 kg (+SD), a height of 65 cm (+SD), and a head circumference of 39 cm (–SD). Neurological examination revealed the patient had poor head control, body hypotonia, limb hypertonia, normal tendon reflex, and no restricted eye movement. Blood biochemical tests, blood lactic acid, and blood ammonia were normal. The patient had severe developmental delay. At 2 months of age, she was able to lift her head, but she lost the ability of head control after the onset. By the time of admission at 4 months of age, she was unable to roll over and pursue objects or sounds. She also showed other neurological symptoms, including irritability, dysphoria, excessive crying, and sleeping disorders. Autonomic symptoms were seen in the patient including ptosis, excessive sweating, and drooling. The family history of the patient was reviewed; the patient's elder brother presented highly similar symptoms at 4 months of age, including paroxysmal eyes deviation, dystonia, developmental delay, and feeding difficulties. He was diagnosed with epilepsy and cerebral palsy. His episodes could not be eased by phenobarbital or sodium valproate, and rehabilitation training for developmental delay had poor improvement. He died at age of 10 years old. The cause of death was reported by the parents as “heart and kidney failure”. However, he did not received genetic tests. No other family members showed similar symptoms.

**Figure 1:**
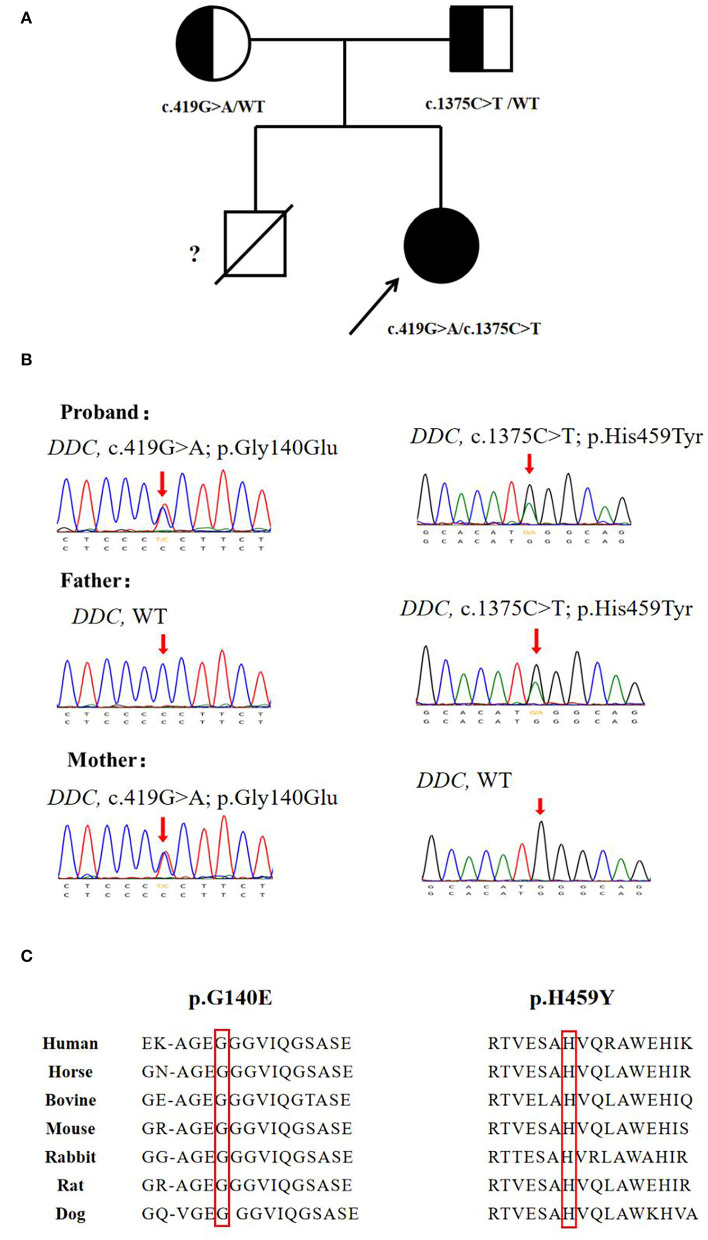
Genetic analysis of the *DDC* gene. **(A)** Pedigree for the proband's family. **(B)** Sanger sequencing analysis for *DDC* mutation identified in the family. **(C)** The missense variants sp.G140E and p.H459Y are highly conserved among species.

Video electroencephalogram (VEEG) during the interictal and ictal periods showed no seizure activity. Head MRI showed delayed myelination. According to the clinical manifestation and EEG results, the episode was determined as an oculogyric crisis with dystonia. Therefore, the possibility of being misdiagnosed as “epilepsy' had risen. Laboratory findings of the patient are shown in Table 1. The results showed elevated 3-O-methyldopa (3-OMD) in dried blood spots. An abnormal pattern of neurotransmitters in CSF was also detected, indicating decreased 3-methoxy 4-hydroxyphenylglycol (MHPG), 5-hydroxyindoleacetic acid (5-HIAA), and homovanillic acid (HVA), together with elevated levels of 3-OMD and 5-HTP, and the methyltetrahydrofolate (5-MTHF) level was normal. The plasma level of AADC was significantly lower than the normal range. Blood amino acid, acylcarnitine spectra, and urine organic acid analysis were negative. The accurate diagnosis of AADCD was confirmed by genetic tests. Medical exome sequencing showed the patient carried novel compound heterozygous mutations in the *DDC* gene:c.419G>A and c.1375C>T, both classified into uncertain significance. Mutation c.419G>A was inherited from her mother, and mutation c.1375C>T was inherited from her father (Figures 1A,B).

**Table 1:** Laboratory findings of the proband.

**Parameter**	**Proband**	**Normal range**
3-OMD(dried blood spot)	2,258.432	6.33–93.28 ng/ml
3-OMD(CSF)	1,660.852	<60 nmol/L
MHPG(CSF)	9.677	52–136 nmol/L
5-HIAA(CSF)	14.136	179–711 nmol/L
HVA(CSF)	79.818	450–1,173 nmol/L
5-MTHF(CSF)	77.778	40–240 nmol/L
5-HTP(CSF)	114.938	<13.69 nmol/L
AADC(plasma)	2.063	16–99 mU/L

3-OMD, 3-O-methyldopa; MHPG:3-methoxy 4-hydroxyphenylglycol; 5-HIAA, 5-hydroxyindoleacetic acid; HVA, homovanillic acid; 5-MTHF, methyltetrahydrofolate; 5-HTP, 5-hydroxytryptophan; AADC, aromatic l-amino acid decarboxylase; CSF, Cerebrospinal fluid.

After the diagnosis of AADCD, the patient was given clonazepam, trihexyphenidyl hydrochloride, and vitamin B6, and gradually treated with selegiline hydrochloride and pramipexole dihydrochloride. The treatment improved her sleeping disorder, and reduced the frequency of oculogyric crisis episodes, every 3–8 days and lasted for 1 h. On the follow-up at 18 months of age, weight and height were in the normal range. The frequency of oculogyric crisis with dystonia attacked every 7–8 days and lasted for 1–2 h. Autonomic symptoms and sleeping problems were improved. However, there was no improvement in her developmental delay. She still had no head control, voluntary movements, or language.

## Discussion

In this case, we reported an AADC deficiency case from a Northern Chinese family whose symptoms mimicked epileptic seizures and were misdiagnosed as epilepsy.

Since the first case described by Hyland (11) in 1990, there have been approximately 200 cases reported worldwide. The mutations reported in AADCD were mainly missense, but the splice mutation c.714 + 4A > T was the most frequent mutation in Southern China ethnicity, possibly due to a founder effect (12). In this case, we reported two novel missense mutations c.419G>A and c.1375C>T. Both of the mutations were not recorded in the genome database gnomAD or the variants database Clinvar and HGMD. The two mutations were calculated as disease-causing by Polyphen-2 and SIFT, and amino acid sequences were highly conserved among species (Figure 1C). Previous biochemical studies on missense mutations have revealed their main pathogenic mechanisms: (1) decreased catalytic efficiency, such as p.R347Q/G and p.L38P; (2) compromised PLP affinity, such as p.G102S, and (3) protein misfolding, such as p.R447H and p.R160W (7). With the increasing number of identified patients, it was found that over 70% of them carried compound heterozygous mutations (13). Biochemical studies discovered that a positive or negative interallelic complementation existed in the AADC dimer. Longo et al. found that the heterodimeric T69M/S147R exhibited negative complementation in accordance with activity, while homodimer T69M reserved 10% of the wild-type catalytic efficiency (13, 14). The finding was consistent with the patients' phenotype: the patients who carried T69M/S147R showed severe symptoms (such as profound hypotonia and dystonia, scoliosis, and inability to walk), and the other patients who carried T69M/T69M presented much milder symptoms (no dystonia and walked independently) (15). The various mutation types (missense, frameshift, and splice site) and the complexity of interactions between homodimer/heterodimeric AADC protein, together, lead to variability in clinical manifestation, ranging from severe to mild. The establishment of genotype-phenotype correlation requires further investigation.

The global prevalence of AADCD remains unclear. Some previous studies have tried to approach it through different methods. Whitehead et al. (16) estimated the prevalence of AADCD to be 1/90,000 in the US, 1/118,000 in the EU, and 1/182,000 in Japan, based on whole-genome or whole-exome sequencing databases. Moreover, Hyland et al. (17) launched the prevalence of AADCD was 1/42,000 after screening biological samples in patients with neurological disorders. Despite the apparent differences, both results indicated that the prevalence of AADCD may be underestimated, indicating many patients may be undiagnosed or misdiagnosed. The clinical characteristics of AADCD were related to the deficiencies of four neurotransmitters directly affected by AADC, which are dopamine, epinephrine, norepinephrine, and serotonin (5). In our case, deficiencies in dopamine can impact cognition and voluntary movement, which may explain the patient's motor developmental delayed and oculogyric crisis and dystonia. The decreased level of serotonin may cause the patient's sleeping disorder and poor appetite. Epinephrine and norepinephrine reduction can explain irritability, dysphoria, and excessive crying. Consequently, the patient's comprehensive deficiencies of neurotransmitters resulted in central nervous system syndromes and autonomic nervous system syndromes, including movement disorders, developmental delay, and behavioral problems.

The consensus summarized the key symptoms of AADCD-included hypotonia, movement disorders, developmental delay, and autonomic symptoms, mostly the onset within the 1st year of life (7). The oculogyric crisis is one of the most common symptoms, observed in nearly 78% (91/117) of the patients with AADCD (1). The episodes of oculogyric crisis show persisted dystonic, conjugate, and typically upward deviation of the eyes, lasting from seconds to hours. Deviation of the eyes can happen isolated or accompanied by involuntary flexions of the face, the neck, the limbs, and even the whole body (18). Oculogyric crises are non-epileptic eye movements, which can be easily confused with tonic eye deviations in epileptic attacks. Pearson et al. reported that 27% (14/52) patients were initially misdiagnosed with epilepsy (6). Furthermore, the cohort study in Mainland China found an even higher misdiagnosed rate of 30.4% (7/23) because of the oculogyric crisis (19). Epilepsy was much less common than the oculogyric crisis in patients with AADCD (20, 21). Previous studies have reported seizures in 9 of 68 cases (1). Ito et al. (20) reported a Japanese patient who manifested epileptic spasms and generalized tonic seizures, together with involuntary non-epileptic movements. Although EEG is not a necessary choice in diagnosing AADCD according to the consensus, it is the only way to make a definite differential diagnosis between oculogyric crisis attacks and epileptic attacks. In addition to epileptic seizures, some hereditary monoamine neurotransmitter disorders show similar oculogyric crisis with AADCD. These disorders include autosomal recessive GTP-cyclohydrolase 1 (GTPCH) deficiency, sepiapterin reductase (SR) deficiency, and tyrosine hydroxylase (TH) deficiency. Since the neurophysiological mechanism of these hereditary disorders is associated with dopamine metabolism, their main clinical features, such as early dystonia, oculogyric crisis, developmental delay, and autonomic dysfunction, are very similar to AADCD. Among these disorders, AADCD is still commonly seen. Analysis of the pattern of neurotransmitters in CSF and genetic tests is the key to differential diagnosis with AADCD (18, 22).

In this case, the proband had typical clinical manifestations of AADCD: developmental delay, hypotonia, and the oculogyric crisis with dystonia. She was classified as a severe phenotype. After 2 months of being misdiagnosed with epilepsy, a video EEG confirmed the episodes were paroxysmal dyskinesia and oculogyric crisis. The diagnosis of AADCD was finally established by the specific pattern of neurotransmitters in CSF, and novel compound heterozygote mutations in the *DDC* gene. The proband's elderly deceased brother was diagnosed with epilepsy and received anti-epileptic treatment. Despite lacking genetic tests or laboratory examinations, his similar symptoms with the proband suggested he was a potential misdiagnosed AADCD patient as well. Therefore, we suggested that pediatric patients with epileptic-like episodes within the 1st year of life should consider AADCD. Another efficient method to early identified patients with AADCD is newborn screening. Chien et al. (23) found dry blood spot screening in newborns for 3-OMD reached a 100% positive-predictive rate to identify patients with AADCD in Taiwan, China.

Distinguishing between epileptic episodes and non-epileptic movements is essential for drug choice. First-line treatment agents are selective dopamine agonists, followed by MAO-inhibitors, and pyridoxine. Additional symptomatic treatment agents are anticholinergic agents, melatonin, benzodiazepines, and Alpha-adrenoreceptor blockers (1, 24). After diagnosis, our patient was treated with clonazepam, trihexyphenidyl hydrochloride, vitamin B6 selegiline hydrochloride, and pramipexole hydrochloride. Her paroxysmal dystonia with the oculogyric crisis was significantly reduced, her sleep improved, and autonomic nervous symptoms were relieved. However, there was no significant improvement in her developmental delay. To prevent complications and improve development, comprehensive treatment, such as physiotherapy, speech therapy, occupational therapy, feeding and nutritional assessment, and psychological treatment, is essential. Gene therapies may bring new insight into patients with AADCD. A few of these therapies are currently under development in research settings (25, 26). Previous research has shown that gene therapy can improve the motor function of individuals with AADC deficiency, and may also improve physiological and cognitive functioning (27, 28). Due to the limited clinical efficacy of drug therapy, gene therapy will be a promising treatment option for children with AADCD.

## Conclusion

In summary, we report a case of AADCD misdiagnosed as an epileptic status. This case emphasized the difficulties and necessity of distinguishing epileptic episodes and oculogyric crisis episodes in patients with AADCD, in which video EEG can help differential diagnosis. Infants with oculogyric crisis and dystonia, developmental delay, and hypotonia should be considered for AADCD. Screening through dry blood spots for 3-OMD should be performed as early as possible. Cerebrospinal fluid neurotransmitter testing, plasma AADC activity testing, and genetic testing should be performed to confirm the diagnosis. Early and accurate diagnosis can avoid unnecessary and incorrect treatment, and improve quality of life.

## Data availability statement

The data presented in the study are deposited in the National Genomics Data Center (NGDC, https://ngdc.cncb.ac.cn/) repository, accession number HRA002290.

## Ethics statement

The studies involving human participants were reviewed and approved by Medical Ethics Committee of Beijing Children's Hospital, Capital Medical University. Written informed consent to participate in this study was provided by the participants' legal guardian/next of kin. Written informed consent was obtained from the individual(s) for the publication of any potentially identifiable images or data included in this article.

## Author contributions

HW, JL, JZ, and WF collected and analyzed clinical information. HW followed up with the patient and contributed to the manuscript. ML and XW analyzed data of EEG. XW contributed to the manuscript. FF, XR, LD, and CD supervised this study. All authors contributed to the article and approved the submitted version.

## Conflict of interest

The authors declare that the research was conducted in the absence of any commercial or financial relationships that could be construed as a potential conflict of interest.

## Publisher's note

All claims expressed in this article are solely those of the authors and do not necessarily represent those of their affiliated organizations, or those of the publisher, the editors and the reviewers. Any product that may be evaluated in this article, or claim that may be made by its manufacturer, is not guaranteed or endorsed by the publisher.
